# Electrowinning of Iron from Spent Leaching Solutions Using Novel Anion Exchange Membranes

**DOI:** 10.3390/membranes9110137

**Published:** 2019-10-24

**Authors:** Wouter Dirk Badenhorst, Cloete Rossouw, Hyeongrae Cho, Jochen Kerres, Dolf Bruinsma, Henning Krieg

**Affiliations:** 1 Focus Area: Chemical Resource Beneficiation, Faculty of Natural Sciences, North-West University, Potchefstroom 2520, South Africa; 24250643@nwu.ac.za (W.D.B.); 22114688@nwu.ac.za (C.R.); J.Kerres@gmx.de (J.K.); 2 Institute of Chemical Process Engineering, University of Stuttgart, D-70199 Stuttgart, Germany; hyeongrae.cho@icvt.uni-stuttgart.de; 3 Bruinsma Solutions, 10 Mclagen Str., Potchefstroom 2531, South Africa; dolf.bruinsma@gmail.com

**Keywords:** electrowinning, anion exchange membranes, waste treatment, iron sulphate disposal, acid regeneration

## Abstract

In the Pyror process, electrowinning (EW) is used to recover acid and iron from spent leaching solutions (SLS), where a porous Terylene membrane acts as a separator between the cathode and anode. In this study, a novel anion exchange membrane (AEM)-based EW process is benchmarked against a process without and with a porous Terylene membrane by comparing the current efficiency, specific energy consumption (SEC), and sulfuric acid generation using an in-house constructed EW flow cell. Using an FAP-PK-130 commercial AEM, it was shown that the AEM-based process was more efficient than the traditional processes. Subsequently, 11 novel polybenzimidazole (PBI)-based blend AEMs were compared with the commercial AEM. The best performing novel AEM (BM-5), yielded a current efficiency of 95% at an SEC of 3.53 kWh/kg Fe, which is a 10% increase in current efficiency and a 0.72 kWh/kg Fe decrease in SEC when compared to the existing Pyror process. Furthermore, the use of the novel BM-5 AEM resulted in a 0.22 kWh/kg Fe lower SEC than that obtained with the commercial AEM, also showing mechanical stability in the EW flow cell. Finally, it was shown that below 5 g/L Fe, side reactions at the cathode resulted in a decrease in process efficiency, while 40 g/L yielded the highest efficiency and lowest SECs.

## 1. Introduction

A South African mining group is investigating the use of electrowinning (EW) to recover iron and H_2_SO_4_ from an iron sulphate waste solution produced during metal beneficiation. For the purpose of this paper, the term electrowinning, as is commonly applied for this process, is used throughout the paper. The spent leaching solution (SLS) consists mainly of iron sulphate, unspent sulfuric acid, and some minor metal impurities such as cobalt (Co), nickel (Ni), and chromium (Cr). Traditionally, such SLSs have been treated using either active or passive methods such as oxidative, bacterial, or limestone precipitation [1,2]. These methods, however, rarely yield marketable products when used to treat the SLSs [1].

The most prevalent method for treatment involves the use of chemical neutralization agents [2]. The addition of a base (NaOH, CaO, or FeCO_3_) raises the pH of the solution, lowering the solubility of many metal species, thereby facilitating the precipitation of metal hydroxides [2]. In the case of iron, oxidizing chemicals such as H_2_O_2_, or non-chemical methods such as active aeration can be used to oxidize Fe(II) into the lower solubility Fe(III), simplifying the precipitation process [2,3,4]. Neutralization, however, produces a large volume of sludge containing only 2–4% solids [2], which must undergo several further processing steps to increase the solid content up to 50% before disposal [2]. 

An alternative and possibly more cost-effective treatment method is EW. EW in an undivided cell, that is, without the use of a membrane separator, is used commercially for the recovery of various metals including copper (Cu), cobalt (Co), and nickel (Ni) [5,6,7]. However, factors that reduce the efficiency of undivided cell EW processes include hydrogen evolution at the cathode and the dissolution of the cathode due to the acid formation in the electrolyte. For higher value metals such as Cu and Co, the reduced efficiency of the process is offset by the value of the product. In such cases, the production of an acid solution at the anode is deemed a waste reaction. For iron recovery, however, with its lower intrinsic value, these factors significantly reduce the profitability and feasibility of the EW process. An additional disadvantage during the EW of iron is the oxidation of Fe(II) to Fe(III) due to oxygen evolution occurring at the anode, reducing the efficiency of the EW process even further [8]. 

Some of the disadvantages associated with iron EW in an undivided cell have been overcome through the use of a porous membrane between anolyte and catholyte, reducing the transfer of oxygen from the anolyte to the catholyte and, subsequently, reducing the amount of oxidized Fe(II) in the catholyte [8]. Additionally, the porous membrane reduces the transfer of H^+^ from the anolyte to the catholyte, thereby reducing hydrogen formation at the cathode, which further increases the current efficiency [8]. An example of using such a porous membrane for the EW of iron is found in the Pyror process (developed during 1947–1957), where iron is recovered from a sulfuric acid environment [8]. In this process, the cathode is inserted into a Terylene bag (porous membrane), with the iron-rich solution being fed to the cathode, wherein the iron is plated. The depleted SLS is transported through the membrane due to a slight overpressure on the feed, where sulfuric acid is produced at the anode by the oxidation of water regenerating H_2_SO_4_ and evolving oxygen. At the anode, remaining Fe(II) is oxidized to Fe(III) in the presence of the oxygen that is formed. Subsequently, the anolyte containing the regenerated H_2_SO_4_ and Fe(III) is fed back to the leaching process, where the Fe(III) is reduced back to Fe(II) in the presence of hydrogen sulfide. The Pyror process was, however, discontinued in the 1950s due to the low intrinsic value of Fe coupled with a decline in the business conditions experienced [8].

The recent advent of non-porous anion exchange membranes (AEMs) that are electrically conductive for anionic species has aided the development of numerous applications, including alkaline membrane electrolysis and redox flow batteries [9]. Initially, most research has focused on applications in basic environments, where the AEMs have to withstand the attack of typically OH- ions on the cationic groups within the AEM [10,11]. More recently, AEMs were used in acidic environments, such as in diffusion dialysis and redox flow batteries. An example of diffusion dialysis entails the recovery of acid from spent pickling solutions containing unspent acid and metal salt mixtures [12,13,14], wherein the unspent acid is separated from the metal species by an AEM that rejects the transfer of cationic metal species whilst allowing transfer of anionic species and protons [12]. Acid-resistant AEMs are additionally used in redox-flow batteries (RFB) such as the all vanadium RFB (VRFB) [15,16]. Here, the AEM prevents the transfer of the vanadium species from the anolyte to the catholyte (preventing unintended discharge) while allowing the transfer of protons and sulphate ions to maintain the charge balance in the electrolytes [15,16]. The AEMs employed in RFB applications are required to be stable in both an acidic and oxidative environment due to the oxidative nature of V^4+^ and V^5+^ [15]. The advantage of using AEMs in VFRBs is that the vanadium cations are excluded from the AEM interior by Donnan exclusion [17], and therefore are unable to attack the membrane polymer from the inside. In contrast, in cation exchange membranes (CEM), where the vanadium ions permeate into the CEM interior, the polymer backbone is degraded much faster, as was shown for sulfonated Radel membranes [18]. However, the use of AEM membranes has not been exhaustively investigated for the notoriously difficult EW of iron from an acidic environment [19], requiring high chemical stability, low cation transfer rate, and low electrical resistance [20,21].

In recent years, a variety of stable AEMs have been developed by blending an anion-exchange polymer or its halo-methylated precursor with a chemically and mechanically stabilizing matrix polymer [22,23,24]. By combining polymers such as polybenzimidazole and a sulfonated cation-exchange polymer, ionic cross-links are formed, further chemically stabilizing the blended AEMs [22,23,24]. In a recent study, a 3-component blended membrane consisting of (i) poly(2,6-dimethyl-1,4-phenylene oxide) (PPO) quaternized with tetra-methylimidazole, (ii) a polybenzimidazole (PBI) as the matrix polymer, and (iii) a sulfonated polymer as ionic cross-linker showing excellent stability and performance in VRFB applications [25]. 

As mentioned previously, AEMs have been used successfully in various electrochemical processes in acidic environments. It was, therefore, the aim of this study to investigate the suitability and stability of AEMs for the EW of iron from an SLS solution. To evaluate the suitability of the EW process for the removal of iron from SLS, the following work was done. Firstly, the AEM-based process for the SLS was benchmarked against, and compared with, the undivided cell and the Terylene process. Subsequently, various novel AEMs were synthesized and evaluated to identify the most suitable membrane in terms of stability, Fe and proton rejection, and electrical conductivity. The performance of the processes and membranes were evaluated in terms of their current efficiency, acid rejection, and specific energy consumption (SEC). Finally, we found the optimum operational window in terms of iron content, the influence of the initial Fe content, as well as the iron depletion on the performance of the AEM-based EW. 

## 2. Materials and Methods 

### 2.1. Flow Cell

**Figure 1:**
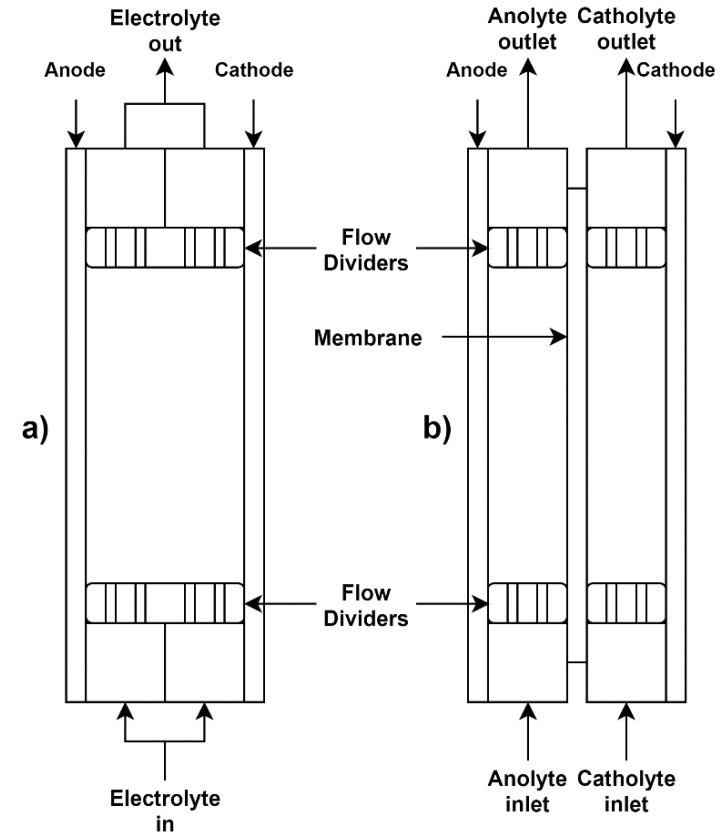
Diagram showing both the unseparated flow cell (**a**) and the membrane separated flow cell (**b**).

For all flow experiments, two custom manufactured EW cells (Figure 1) were used, one without a membrane and one with a membrane. Both cells were constructed from 10 mm thick polycarbonate sheets with an anode–cathode distance (ACD) of 20 mm. For the undivided cell (Figure 1a) no membrane was present. For the divided cell, either a Terylene porous membrane was used to simulate the EW used during the Pyror process, or an AEM was used. The anode (half-reaction: H_2_O → ½O_2_ + H^+^ + 2e^−^) was constructed of 2 mm thick lead doped with 0.06% Cu (Castle Lead Works). The lead plate was passivated by forming PbO_2_ on the surface of the plate through EW of a stock solution of 40 g/L Fe (using FeSO_4_·7H_2_O, ACE Chemicals, 99%) and 60 g/L Na_2_SO_4_ (ACE Chemicals, 99%) catholyte with a 60 g/L Na_2_SO_4_ anolyte for 5 h [26]. The cathode starter plates (half-reaction: Fe^2+^ + 2e^−^ → Fe_s_) consisted of 316 stainless steel plates, with the plated deposits being removed after each 5 h run in order to keep the ACD constant throughout testing. The flow rate of both the catholyte and anolyte (or electrolyte in the case of the undivided cell) was adjusted to 1 cm/s superficial velocity parallel to the electrode and membrane surface. The relatively high flow rate was chosen to reduce the concentration gradient over the electrode and membrane surface area. In addition to this, the cell was fitted with inlet and outlet flow dividers to prevent the formation of dead zones over the cathode and anode, which was confirmed using a dye solution.

The temperature of the electrolytes was kept at 70 °C using a water bath. All the experiments were conducted at a constant current density of 300 A/m^2^, which implies a current of 3 A being applied to the 0.01 m^2^ electrode area (10 × 10 cm^2^ plates). The constant current was applied using a galvanostatic power source (manufactured by the electrical engineering faculty of the North-West University) by regulating the applied voltage. The voltages were recorded at hourly intervals to determine the specific energy consumption (SEC) of the process.

Then, 20 mL samples were taken hourly for 5 h from the electrolytes, with the first sample being taken before the potential was applied. The Fe content in the samples was analysed using inductively coupled plasma optical emission spectroscopy (ICP-OES, Agilent 5110). Furthermore, the pH of the samples was measured using a Metrohm 774 pH meter with an Unitrode 6.0258.010 electrode. To determine the amount of iron plated after 5 h, the cathode was weighed before and after each experiment. The change in mass was then used to determine both the current efficiency and the SEC of the process together with the voltage. The current efficiency was calculated using Equation (1).

(1) $$\mathrm{Current}\;\mathrm{efficiency}\;\left(\%\right)=\frac{\left(m_{f}-m_{i}\right)}{\left(\frac{\mathrm{It}}{\mathrm{nF}}\right)M}\times 100\%$$

where $m_{f}$ and $m_{i}$ refer to the final and initial cathode weight respectively (g), I is the applied current (A), t is the duration (s), n is the number of electrons transferred, F is Faradays constant (96.485 sA/mol), and M is the molar mass of Fe (g/mol). Similarly, the SEC expressed in kilowatt-hours per kilogram of iron plated was determined using Equation (2), where V is the applied potential (V) and m the total mass plated on the cathode (g).

(2) $$\mathrm{SEC}\;\left(\mathrm{kWh}/\mathrm{kg}\right)=\frac{\mathrm{IV}}{m/t}\times 3600$$

The standard electrowinning conditions that were used in this study have been summarized in Table 1.

As mentioned previously, this study had three objectives, (i) benchmark the non-porous AEM-based EW process by comparing it to an EW process without a membrane and one with a porous Terylene membrane, (ii) develop and test novel polybenzimidazole-based AEMs for the EW of iron from an SLS, and (iii) determine the influence of the Fe content on the current efficiency.

### 2.2. EW Cell Configuration

#### 2.2.1. Undivided

For the EW without a membrane, using the setup from Figure 1a, 2L of electrolyte was prepared by dissolving the appropriate amount of FeSO_4_·7H_2_O (ACE Chemicals, 99%) in deionised water to obtain a 40 g/L Fe solution. Additionally, 60 g/L Na_2_SO_4_ (ACE Chemicals, 99%) was added as well as 10 g/L H_3_BO_3_ (ACE Chemicals, 99.5%) acting as a pH buffer (pKa of 9) [6,27,28].

#### 2.2.2. Porous Membrane

When operating the EW cell with a porous membrane, the DP580 Terylene-membrane, supplied by Clear Edge Filtration SA (Pty) Ltd, was used to separate the cathode and anode (Figure 1b). While the flows of the catholyte and anolyte were kept constant at a superficial velocity of 1 cm/s, the catholyte tank was raised by ±10 cm compared to the anolyte vessel to provide a slight head on the catholyte compartment. This resulted in the slow permeation of the catholyte into the anolyte solution at a rate of approximately 2.5 mL/min. Similar to the undivided cell study, the catholyte consisted of a 1 L solution containing 40 g/L Fe (as FeSO_4_·7H_2_O), 60 g/L Na_2_SO_4_ (supporting electrolyte) [8], and 10 g/L H_3_BO_3_, whereas the 1 L anolyte solution contained only the 60 g/L Na_2_SO_4_ as supporting electrolyte and 10 g/L H_3_BO_3_ as buffer.

#### 2.2.3. Anion Exchange Membrane

For testing the AEMs, the same configuration as for the testing of the Terylene membrane was used (Figure 1b). The same amount and composition of catholyte and anolyte from the Terylene setup were used. For the benchmarking a commercial AEM (FAB-PK-130, ion exchange capacity (IEC)—0.7 meq/g, water uptake—15%) was obtained from Fumatech GmbH. The AEM was pre-treated in a 0.5 M NaCl solution at 25 °C for 24 h before being used in the EW cell. Again, a superficial flow rate of 1 cm/s was used.

### 2.3. AEM Synthesis, Characterisation, and Screening

#### 2.3.1. Membrane Synthesis

After comparing the three processes, novel AEMs were developed and tested. For this purpose, 11 different blend membranes were synthesised. The membranes MHC4-C, 2281A, 2285A, 2244B, 2244C, 2252A, BM-5, and 2408-2 were prepared from a halogenated polymer, a PBI polymer, a sulfonated polymer, and 1,2,4,5-tetramethyl-imidazole (TMIm). The membranes 2243A, 2258, and 2259 additionally contained a polyethylene glycol diepoxide, reacting with the N–H groups of the polybenzimidazole blend component to form covalent bonds, providing increased alkali stability [24]. The compositions of the membranes are given in Table 2, whereas Table 3 provides the structures of the various components used. The membranes were prepared by dissolving and combining the halogenated PBI and sulfonated polymer components in dimethyl sulfoxide (DMSO) as described previously [24]. Subsequently, TMIm and poly(ethylene glycol diepoxide) (PEG-diepoxide) for the membranes 2243A, 2258 and 2259) was added to the blend solution. The mixture was then poured onto a level glass plate and spread evenly over the surface. This was followed by the evaporation of the DMSO in a Thermo Scientific Heratherm advanced protocol oven for 2 h at a temperature of 150 °C. During solvent evaporation the reaction of the TMIm with the halomethyl groups (quaternization of the imidazole to tetramethylimidazolium anion-exchange group) took place. After solvent evaporation, the membranes were removed from the glass plate by immersion in water, and subsequently placed in a 10% sodium chloride (NaCl) solution for 24 h at 90 °C, after which they were placed in deionised water for a further 24 h at 60 °C. Before use, the membranes were pre-treated by immersing them in a 0.5 M NaCl solution at 25 °C for 24 h. For membranes 10 and 11 the same process was followed, however, they were cast on a glass fibre cloth (18 g/m^2^, hobbyking) to increase the mechanical strength of the membranes, due to expected concern regarding mechanical strength when employing the AEMs in the EW cell.

#### 2.3.2. Membrane Characterisation

After synthesis, the membranes were characterised by determining their ion exchange capacity (IEC), conductivity (σ), and water uptake (WU %). To determine the IEC, the membranes were immersed in a 1 M KOH solution for 24 h at 90 °C, converting the AEM from the chloride form to the hydroxide form. Subsequently, the membranes were washed with deionised water to remove excess hydroxide from the membrane, before being immersed for 24 h in a 60 mL saturated sodium chloride solution to exchange the anion-exchange groups with the chlorides. Then, 3 mL of a standard 0.1 N hydrochloric acid solution was added to the saturated sodium chloride solution and kept overnight. The membranes were then washed with 25 mL deionized water, and this water was added to the 60 mL saturated sodium chloride solution. Titration was performed with a 0.1 N sodium hydroxide solution. Finally, the membranes were washed several times using deionized water and placed in an oven where they were dried for 24 h at 130 °C. The total IEC was calculated according to Equation (3),

IEC = (C_HCl_ V_HCl_ − C_NaOH_ V_NaOH_)/m_dry_ (3)

where IEC is ion exchange capacity (mmol/g), C_HCl_ is concentration of a hydrochloric acid solution (M), V_HCl_ is used volume of the hydrochloric acid solution (mL), C_NaOH_ is concentration of the sodium hydroxide solution (M), V_NaOH_ is added volume of the sodium hydroxide solution (mL), and m_dry_ is dry weight of the membrane (g). 

The conductivity of Cl^−^ ($\sigma_{{\mathrm{Cl}}^{-}}$) through the AEMs was determined using a Zahner-elektrik IM6 impedance spectrometer. The impedance was recorded at room temperature in 1M NaCl solution with a frequency range of 200 KHz to 8 MHz in the potentiostatic mode (amplitude 10 mV). Membrane resistance was calculated from the intercept in the real axis. From this, the conductivity was determined using Equation (4), where σ is conductivity (mS/cm), R_sp_ is resistivity (Ω cm), d is thickness of membrane (cm), R is ohmic resistance (Ω), and A is electrode surface (cm^2^).

σ = 1/R_sp_ = d/RA (4)

The water uptake (WU) was determined by weighing the dry membrane samples, followed by the soaking of the membranes in deionised water at 25 °C and 90 °C for 24 h. Before determining the wet weight, the surface of the membrane was blotted with tissue paper to remove excess water from the surface. Water uptake was calculated using Equation (5). 

WU (%) = (Wet weight − Dry weight)/Dry weight × 100%(5)

To investigate the thermal stability of the membranes, thermal gravimetric analysis (TGA) was done by heating at a rate of 20 °C per minute under O_2_/N_2_ atmosphere (65–70 °C) using a NETZSCH TGA, model STA 499C.

#### 2.3.3. EW Screening of Novel AEMs

The same setup described previously (Figure 1b) was used with the same anolyte and catholyte composition discussed for the benchmarking study. Membranes were pre-treated in 0.5 M NaCl solution at 25 °C for 24 h before they were used in the EW cell.

## 3. Results

As stipulated in the objectives of this study, the AEM-based EW of iron was first benchmarked against two existing processes, followed by the evaluation of novel PBI-based blend membranes for EW. Finally, the influence of the initial Fe content and its depletion on the performance of the AEM based-EW is discussed. 

### 3.1. EW Cell Configuration

#### 3.1.1. Undivided

**Figure 2:**
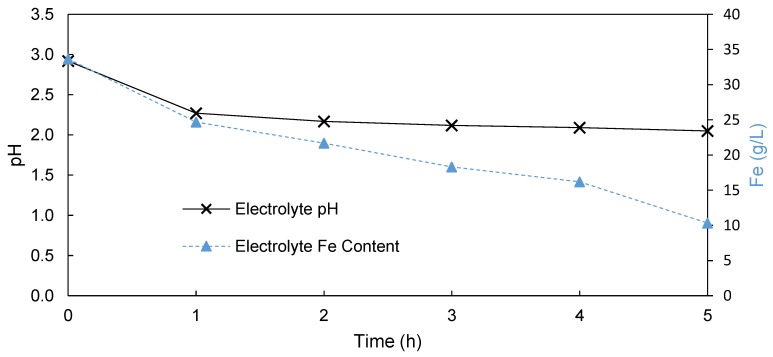
Change in the pH (primary *y*-axis) and Fe content (secondary *y*-axis) of the electrolyte during electrowinning (EW) in an undivided cell.

Figure 2 shows the change in pH and Fe content of the electrolyte during the EW process in the absence of a separator between the cathode and anode. The pH started at a value of 2.92, decreasing to 2.27 and 2.05 after 1 and 5 h, respectively. This indicated the successful production of H_2_SO_4_. It can be noted, however, that after the initial significant increase in acid content between 0 and 1 h, the rate of acid production thereafter declined significantly. This implies that after 1 h the acid formed on the lead anode was most probably consumed by the production of H_2_ in the presence of the increased H^+^ content at the cathode, confirming the need of a separator.

Figure 2 also shows the change in the Fe content in the electrolyte, which decreased near linearly over the duration of this experiment. As the Fe content in solution declined by 23.34 g/L, 3.30 g of Fe was plated on the cathode. The mass balance discrepancy was accounted for by the oxidation of the Fe(II) into the less soluble Fe(III) according to Equation (6), which then precipitated as ferric oxyhydroxide at the relatively high pH of the electrolyte (pH = 2–3) [3,4]. The precipitate was identified as goethite/ferric oxyhydroxide using XRD analysis (data available in the Supplementary Materials section).

(6) $$2{\mathrm{Fe}}^{2+}+\frac{1}{2}O_{2}+3H_{2}O\;\to 2\mathrm{Fe}\left(\mathrm{III}\right)O\left(\mathrm{OH}\right)+4H^{+}$$

Due to these side reactions, which can be attributed to the absence of a divider between the anode and the cathode, the undivided cell process resulted in a current efficiency of only 21%. This led to a high SEC of 17 kWh/kg Fe, which makes this process not feasible for the commercial processing of SLS. The presence of the precipitated ferric oxyhydroxide also complicates the industrial process due to the degradation of rotating equipment. Considering the low efficiency, high SEC, and precipitation that occurred, this undivided cell process is not feasible for the EW recovery of iron and the production of H_2_SO_4_.

#### 3.1.2. Porous Membrane

**Figure 3:**
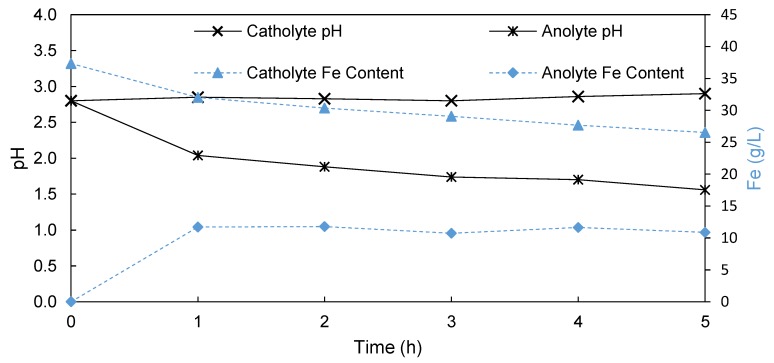
Change in the pH (primary *y*-axis) and Fe content (secondary *y*-axis) of the catholyte and anolyte during EW using a Terylene porous membrane as a separator.

In Figure 3, the change in pH and Fe concentration in solution of both the catholyte and anolyte is presented when using a porous Terylene membrane to separate the electrolyte into a catholyte and anolyte. The pH of the catholyte increased from 2.80 to 2.90 during the 5 h run, confirming that no significant leakage of protons produced on the anode to the catholyte had occurred. This implies that the slight overpressure applied on the catholyte solution was adequate in restricting the proton movement from anolyte to catholyte. This was also confirmed by the absence of bubbles forming at the cathode during the run. Simultaneously, the anolyte pH decreased from 2.80 to 1.56 over 5 h, which was significantly lower than that obtained during the EW in the undivided cell (pH = 2.05). Accordingly, this indicates that the Terylene-based porous membrane process yielded an improved production of spent H_2_SO_4_ from the SLS compared to that of the undivided cell.

During EW using a porous Terylene membrane, the amount of iron plated increased from 3.30 g when using the undivided cell process to 12.10 g. Also visible in Figure 3 is the decrease in the dissolved iron concentration in the catholyte, which was less than that observed in the undivided cell process. This implies that the amount of iron lost due to ferric oxyhydroxide precipitation was significantly reduced, as the iron was no longer in direct contact with the oxygen producing anode. Some transfer of iron from the catholyte to the anolyte was observed as a result of the porous nature of the Terylene membrane, and the slight overpressure applied. However, due to the suppression of both the hydrogen formation and iron oxidation side-reaction, the current efficiency increased from 21.10% in the undivided cell process to 77% when using the Terylene membrane. As a result of the increased current efficiency, the SEC lowered from the 17 kWh/kg Fe in the undivided cell process to only 9.5 kWh/kg iron when using a Terylene membrane. This confirms that the Terylene process has the capability of removing more iron from the SLS at higher efficiencies and lower SECs when compared to the undivided cell, confirming its use in the Pyror process. 

#### 3.1.3. Anion Exchange Membrane

**Figure 4:**
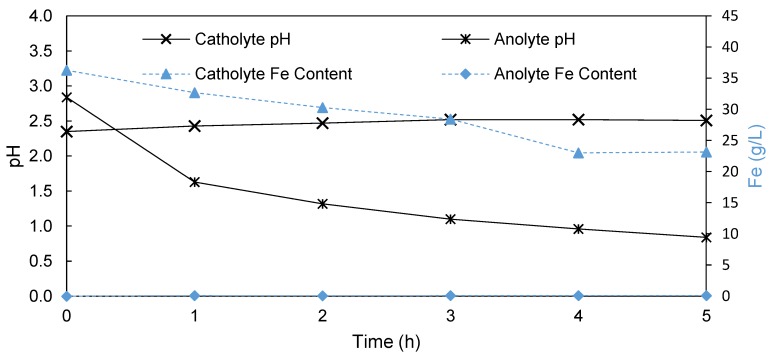
Change in the pH (primary *y*-axis) and Fe content (secondary *y*-axis) of the catholyte and anolyte during EW using an AEM (FAB-PK-130) as a separator.

In an attempt to further reduce both the iron as well as proton transport, a non-porous AEM (FAB-PK-130, Fumatech GmbH, Bietigheim-Bissingen, Germany) was used to separate the catholyte and anolyte. Again, the change in both the pH and Fe content in the anolyte and catholyte was monitored over a 5 h period. According to Figure 4, the constant increase in the catholyte pH from 2.35 to 2.51 in 5 h was similar to the trend observed with the Terylene membrane, showing little proton leakage into the catholyte. However, using an AEM, a more significant drop of the anolyte pH after 5 h (pH = 0.84) compared to that of the Terylene process (pH = 1.56) was observed. This confirms a higher proton rejection by the AEM membranes, confirming their suitability for this process.

Although the iron content decreased from 36.3 g/L to 23.2 g/L in 5 h in the catholyte, the iron content in the anolyte increased to only 0.1 g/L over 5 h (Figure 4), compared to the 10.9 g/l found in the anolyte after 5 h when using Terylene, confirming that the AEM prevented the transport of iron across the membrane. Furthermore, the AEM outperformed both the EW cell with and without a membrane in terms of the amount of iron plated. During the AEM-based EW, 15.5 g of iron was plated onto the cathode during the 5 h run. This resulted in a current efficiency of 99%, which is a significant 22% increase over the Terylene-based EW process. Accordingly, the SEC decreased to 3.75 kWh/kg iron plated compared to the SEC of 9.54 kWh/kg iron when using a Terylene membrane. This implies that the AEM outperformed the reported values for the previously reported Pyror process, both in terms of the current efficiency (14%) and SEC (0.5 kWh/kg) [8]. 

Table 4 summarises the most important data obtained with the three EW processes. It is clear that the division of the cell using either a Terylene or an AEM resulted in fewer side reactions (H_2_ produced at the cathode and iron oxidation due to O_2_) leading to lower pH values and less Fe precipitation as ferric oxyhydroxide. Additionally, a sharp increase in current efficiencies was observed when EW iron had a separator—the efficiency improved with Terylene by 56.21% and with an AEM by 77% compared to operation of the EW cell without a separator. Similarly, the SEC decreased from 17.7 to 3.75 kWh/kg when comparing the undivided and AEM-based processes. It is clear that the AEM process yielded the most iron with the highest efficiency, lowest SEC, and most acid produced.

### 3.2. AEM Synthesis, Characterisation, and Ccreening

#### 3.2.1. Characterisation

After the successful preparation of the 11 novel blend membranes (Table 3), the membranes were characterised in terms of their IEC_total_, Cl^-^ conductivity, and water uptake (WU) at 25 °C and 90 °C, the results of which are presented in Table 5. The IEC of all the membranes were in the range of 2.45 to 2.89 meq/g, which is a suitable range for AEM applications requiring an IECs > 2 for a successful application in a membrane process [23,25]. The chloride conductivity varied significantly, ranging from 1.6 to 45.5 mS/cm between the various membranes. This was, however, the ex-situ chloride conductivity, with the actual membrane conductivity being different during the EW process. In contrast to this, membranes 9 and 11 showed a lower IEC and chloride conductivity, which was ascribed to the formation of increased covalent bonds in the blend system. The water uptake for most membranes was in the range of or less than 50%, whereas 2258 approached 100% (at 90 °C) and 2259 ranged between 160% (at 25 °C) and 260% (at 90 °C). This higher WU of the two mentioned membranes can be ascribed to the high IEC and low cross-linking density, and to the hydrophilic PEG content of the 2258 and 2259 membrane [24], respectively. 

During the TGA analysis of the membranes (2258, 2259, 2244B, 2244C, 2252A, 2281A, 2285A, data shown in Supplementary Information), showed an initial slight mass loss between 100 °C and 200 °C, which can be attributed to the loss of water from the membrane. Degradation for all the membranes only started above 250–260 °C, confirming that their thermal suitability for an EW process operated at 70 °C. 

#### 3.2.2. EW Screening of Novel AEMs

Some of the novel AEMs seemed to lack mechanical stability during the 5 h EW run. Membranes (1) 2243A, (2) MHC4-1C, (3) 2258, (4) 2281A, and (5) 2285A showed immediate damage, indicated by a significant transfer of ions across the membrane, leading to mixing of the catholyte and anolyte (images of membranes 2243A (1) and MHC4-C (2) are shown in the Supplementary Materials section, the other membranes showed similar failure). Because no experimental data could be obtained for these membranes, they are not discussed further in this section. Apart from the membranes failing immediately, membranes (8) 2244C and (9) 2252A failed after 3 and 2 h, respectively, with similar damage noted as with membranes 2243 (1) and MHC4-C (2). In their case, the results obtained in that period were used to calculate the current efficiency and SEC after said time. Although membranes (6) 2244B and (7) 2259 displayed some visual deformation over the 5 h experiment, these membranes remained functional over the duration of the run by successfully separating the catholyte and anolyte. This was in spite of the substantial water uptake measured for membrane 2244B in the previous section. 

The performance of the membranes that did not fail immediately (6–11) will be discussed in terms of the anolyte and catholyte pH, the iron concentration anolyte, the current efficiency, the SEC, and the membrane lifetime. For comparative purposes, the results obtained when using the commercial FAB-PK-130 membrane were included.

**Figure 5:**
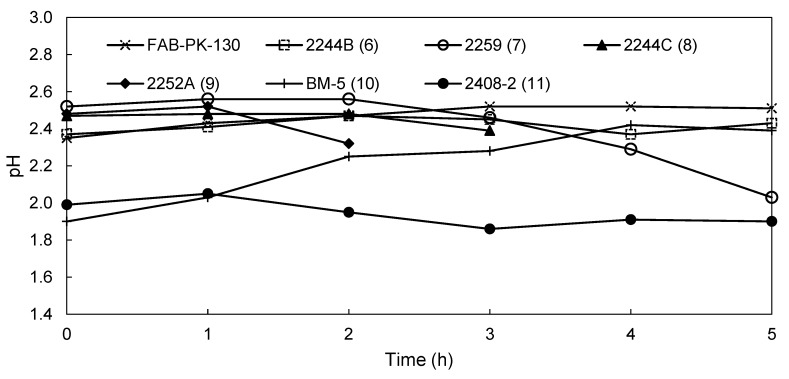
Change in the catholyte pH for membranes FAB-PK-130, 2244B (6), 2259 (7), 2244C (8), 2252 A (9), BM-5 (10), and 2408-2 (11) during EW.

Figure 5 shows the pH of the catholyte solutions of the novel membranes and the commercial FAB-PK-130 membrane as a reference. Both the FAB-PK-130, 2244B (6), and BM-5 (10) membranes showed a slight increase in the pH of the catholyte, with the catholyte pH of the 2244B (6) membrane (pH = 2.43) being slightly below that of the FAB-PK-130 membrane (pH = 2.51), suggesting slightly higher H^+^ transport across the membrane from the anolyte to the catholyte. Additionally, the BM-5 (10) membrane started at a low initial catholyte pH of 1.9 but ended with a catholyte pH of 2.39 just below the benchmark FAB-PK-130 membrane (pH of 2.51). For the other membranes, a decline in pH occurred after 1 h for the 2252A (9) and 2408-2 (11) membranes. Furthermore, the decline in pH occurred for membranes 2259 (7) and 2244C (8) after 2 h. This coincided with an observed increase in H_2_ gas formation on the cathode during the run. It is, however, difficult to ascertain whether this increase was due to chemical or physical degradation.

**Figure 6:**
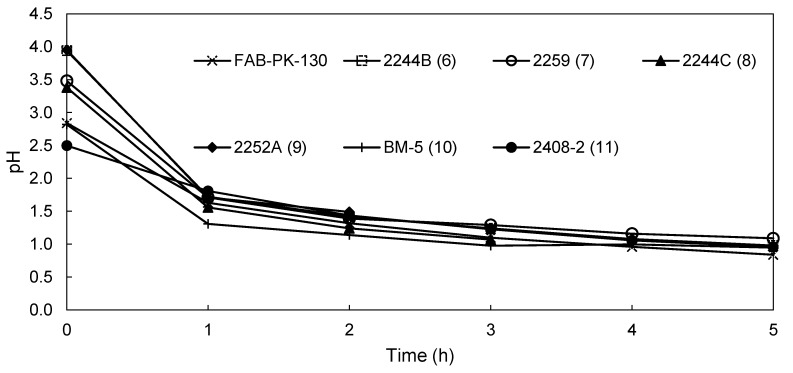
Change in the catholyte pH for membranes FAB-PK-130, 2244B (6), 2259 (7), 2244C (8), 2252 A (9), BM-5 (10), and 2408-2 (11) during EW.

In Figure 6, the pH of the anolyte of the novel membranes and the commercial FAB-PK-130 membrane is shown. In line with the catholyte pH (Figure 5), the FAB-PK-130 yielded the lowest pH after 5 h, reaching 0.84 compared to the 0.98 and 1.09 obtained by 2244B (6) and 2259 (7), respectively, with the two membranes with glass support (BM-5 (10) and 2408-2 (11)) obtaining end anolyte pH values of 0.95 and 0.96, respectively. After 3 h, that is, before failing, 2244C (8) performed marginally better than the commercial membrane after the same amount of time. The tendencies, however, for all the membranes were similar, showing a significant decline in pH after the first hour with a smaller and linear decline thereafter.

**Figure 7:**
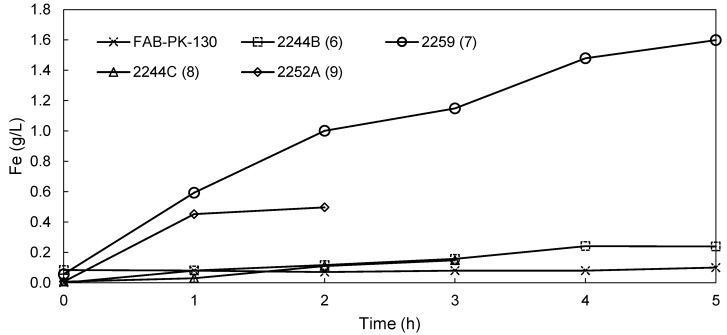
Change in the anolyte Fe content for membranes FAB-PK-130, 2244B (6), 2259 (7), 2244C (8), and 2252 A (9) during EW.

It is shown in Figure 4 that no iron had crossed to the anolyte over a 5 h period when using the FAB-PK-130. For comparison, the Fe transfer to the anolyte for the novel AEMs is shown in Figure 7, where the data of FAB-PK-130 was again included for comparison. It is clear that here the most significant differences in the performance of the various membranes was observed. FAB-PK-130 displayed the lowest Fe cross-over (0.1 g/L iron transported in 5 h), followed by 2244B (6) and 2244C (8) with 0.24 g/L (5 h) and 0.15 g/L (3 h), respectively. This is in line with the results of both the anolyte and catholyte pH changes. In contrast, the 2244B (6) membrane had nearly transported 1.6 g/L iron to the anolyte after 5 h with a near-linear transport rate over time. It is interesting to note that this did not significantly influence the pH that was attained in the catholyte (Figure 6). However, the iron transport was in line with the decline observed in the anolyte pH (Figure 5), indicative of increased proton transport across the membrane. This seems to suggest that over time the transport of both anions and cations increased when using membrane 2244C (8), suggesting chemical degradation during the testing.

Table 6 again provides a summary of the data obtained during the EW of the stable PBI-based blend membranes (6–11) in comparison to the commercial FAB-PK-130 membrane. It is clear that over 5 h, the commercial FAB-PK-130 membrane had the best overall performance, followed by 2244B (6) and 2259 (7). However, before failure, 2252A (9) showed a current efficiency and SEC comparable and even slightly higher than that obtained with the FAB-PK-130 membrane at that specific time. On the other hand, apart from failing after 3 h, the 2252A (9) membrane showed a relatively high proton as well as Fe transfer rate, which reduced the suitability of this membrane. During testing of the reinforced membranes BM-5 (10) and 2408-2 (11), they performed well with current efficiencies of 95% and 88%, respectively. It is of note that the BM-5 (10) membrane, despite its lower current efficiency compared to the FAB-PK-130 (99%), operated at a lower SEC of 3.53 kWh/kg Fe compared to the 3.75 kWh/kg Fe of the FAB-PK-130. This was attributed to the lower applied voltage experienced when employing the BM-5 (10) membrane over the FAB-PK-130, which decreased the SEC.

### 3.3. Influence of Fe Content on AEM-EW Process

**Figure 8:**
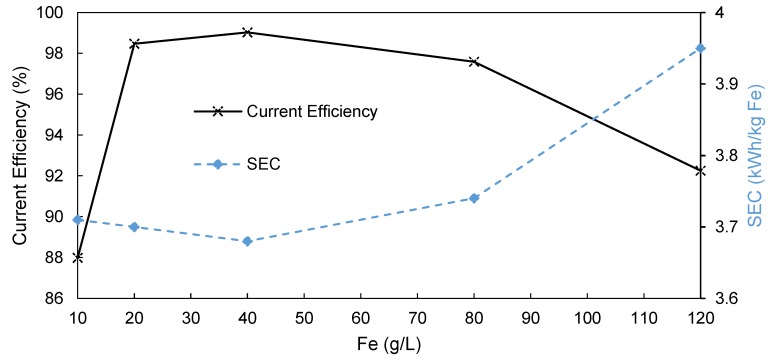
The effect of the initial iron concentration of the catholyte on the current efficiency (primary *y*-axis) and the specific energy consumption (secondary *y*-axis) when using a FAB-PB-130-divided EW cell.

The second objective of the study was to determine the effect of both the initial Fe content as well as Fe depletion on the overall performance of the EW process when using an AEM. Figure 8 shows the dependence of both the current efficiency and the SEC on the initial iron concentration in the catholyte. The highest and therefore optimal current efficiency of 99% was obtained at 40 g/L iron. Below 40 g/L, the efficiency dropped slightly to 98% at 20 g/L iron before dropping significantly to 88% at 10 g/L. This drop could be due to the occurrence of side reactions, as the reducible iron in solution started to deplete around the cathode. An expected side reaction entails the reduction of water according to Equation (7) [29]. 

(7) $$2H_{2}O+2e^{-}\to {\;H}_{2}+2{\mathrm{OH}}^{-}-0.83V$$

Above 40 g/L, the efficiency again decreased slightly to 97.6% at 80 g/L, declining further to 92.3% at 120 g/L. This was likely due to the parasitic reaction of Fe(III) at the cathode, shown in Equation (8) [29], because the FeSO_4_ used to prepare the solutions contained approximately 1% Fe(III), and increasing the iron content in the solution increased the amount of Fe(III) in the solution. Given that Fe(III) is a much stronger oxidizing agent than Fe(II) [29], the reduction of Fe(III) to Fe(II) occurred with more ease at the cathode compared to the reduction of Fe(II) to Fe_s_ (Equation (9)) [29]. 

(8) $${\mathrm{Fe}}^{3+}+e^{-}\to {\;\mathrm{Fe}}^{2+}+0.77V$$

(9) $${\mathrm{Fe}}^{2+}+2e^{-}\to {\;\mathrm{Fe}}_{\left(s\right)}-0.44V$$

The SEC changes (Figure 8) with changing initial Fe content were similar but inverse to the current efficiency data discussed above, that is, reaching a minimum at 40 g/L and declining with both increasing and decreasing Fe content. It is, however, interesting that the SEC increase between 10 and 20 g/L was not as significant as the decrease in current efficiency in this region. 

**Figure 9:**
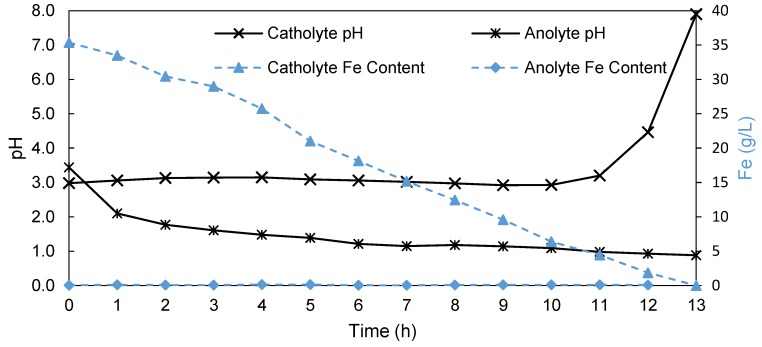
The pH (primary *y*-axis) and iron content (secondary *y*-axis) of both the catholyte and the anolyte over the depletion run.

It was shown in Figure 8 that the reduction of the Fe content, especially below 20 g/L, significantly influenced the current efficiency specifically. Simultaneously, the aim of an EW process to recover iron from an SLS is to reduce the iron content as far as possible. It is therefore imperative to determine the effect of depleting iron content of the process variables. In Figure 9, the change in both pH and iron content of both the catholyte and anolyte is shown over the duration of the depletion run (13 h), using the commercial FAB-PK-130 membrane starting with a 35 g/L iron concentration in the catholyte. 

As previously shown (Figure 4), the anolyte pH decreased continuously, in this case (Figure 9) starting at a pH of 3.44 and ending at a pH of 0.88 after 13 h, which corresponded to approximately 12.9 g/L H_2_SO_4_ that was produced. In contrast, the catholyte pH increased slightly, reaching a pH of 3.15 after 4 h. Between 4 and 10 h, the pH declined slightly to 2.93 after 10 h. This suggests the possibility of slight proton leakage from the anolyte into the catholyte as the anolyte pH decreased. However, after 10 h, the catholyte pH increased exponentially, reaching a pH of 7.9 after 13 h. This supports the earlier discussion on the reduction of water (Equation (7)), occurring when the iron concentration becomes too low. It is interesting to note that the acid formation at the anode remained unaffected by the initiation of side reactions at the cathode, confirming the integrity of the membrane after 13 h.

The catholyte Fe content declined steadily over the 13 h period, decreasing from 35 g/L iron to 0.004 g/L iron after 13 h. This shows that in using a divided EW cell, together with the FAB-PK-130 membrane, approximately 99.99% of the iron can be removed from the initial solution. However, if side reactions at the anolyte should be avoided, which became significant after 10 h, that is, below 6.4 g/L Fe, it seems that a concentration of ±5 g/L Fe is the minimum attainable without side reactions and the corresponding loss in current efficiencies. During the 13 h, the FAB-PK-130 showed hardly any transport of iron from the catholyte into the anolyte, with only 0.06 g/L iron present in the anolyte after 13 h compared to the 0.05 g/L found initially. For the 13-hour run, the current efficiency was 93.04% with an SEC of 3.71 kWh/kg. Although this value is less efficient than what had been obtained after 5 h (higher Fe content), this AEM-based EW process is still 8% more efficient with an SEC 0.54 kWh/kg lower than the Pyror process [8]. The deviation from the previously achieved 99% current efficiency, using the AEM as a divider in the EW cell, was likely caused by the occurrence of side reactions during the last 2 h of the run, that is, when reducing the iron content from 4.43 g/L iron to 0.004 g/L.

## 4. Conclusions

During this study, three different EW methods for the recovery of iron and regeneration of H_2_SO_4_ from an SLS were investigated: (i) EW without a separator, (ii) EW with a porous membrane, (iii) EW with an AEM. 

The AEM process with a FAB-PK-130 membrane outperformed both the undivided and porous membrane process, yielding a current efficiency of 99% and an SEC of 3.75 kWh/kg, which is a 78% and 22% increase, respectively, in current efficiency compared to the undivided and porous membrane process. The high performance of the AEM process is attributed to the high acid rejection and oxygen rejection properties of the AEMs, significantly reducing the number of side reactions. Furthermore, the AEM process described in this paper outperformed the previously established Pyror process for SLS treatment, yielding 14% higher current efficiencies and a 0.5 kWh/kg lower SEC.

During the testing of the novel AEMs, two of the membranes showed promising results, with membranes 2244C and 2252A yielding a current efficiency of 83% and 99%, respectively. However, both these membranes lacked sufficient mechanical strength failing after 3 and 2 h, respectively. In contrast, membranes 2259 and 2244B were mechanically stable (5 h), but only yielded current efficiencies of 70% and 72%, respectively. In contrast to the unsupported membranes, the membranes that were cast onto glass fiber mats (BM-5 and 2408-2) showed high current efficiencies, low SEC values, and mechanical stability. The BM-5 (10) membrane operated at a SEC of 3.53 kWh/kg Fe, which was 0.22 kWh/kg Fe lower than the FAB-PK-130, membrane despite its lower current efficiency of 95%.

The optimal iron concentration was found to be 40 g/L, yielding an efficiency of 99% with an SEC of 3.75 kWh/kg. Below 20 g/L, a decline in the current efficiency was observed, dropping from 99% at 40 g/L iron to 88% efficiency at 10 g/L. From the depletion experiment, it was shown that this process required 13 h to remove 99.99% of the iron from the SLS at a current efficiency of 93% with an SEC of 3.71 kWh/kg. This again confirms the advantage of this process over the Pyror process which, due to its design, is not able to remove the majority of the iron from the SLS [8].

This study has shown that an AEM-based process is suitable for the EW of iron, improving on the previously applied methods. Additionally, the novel AEMs synthesized were chemically suitable for the EW of iron; however, they required additional mechanical support. Membranes BM-5 (10) and 2408-2 (11) that were cast onto the glass fiber showed both mechanical and chemical stability. To improve the performance of AEMs for the iron electrowinning process further, the following work is envisaged: (i) introducing different mechanical reinforcements to the membranes using appropriate supports such as expanded polytetrafluoroethylene (PTFE, ePTFE) foils, or electro spun fiber mats; (ii) improving the synthesized AEMs by developing AEMs derived from partially fluorinated polymers while introducing sterically hindered, that is, bulky cationic solid ions into the polymers; and (iii) to determine the influence of possible impurities that might be present in the real process.

## Figures and Tables

**Table 1:** Standard electrowinning conditions.

Variables	Unit	Value
Membrane/electrode area (A)	cm^2^	100 (10 × 10)
Anode cathode distance (ACD)	cm	2
Cell constant (ACD/A)	cm	50
Electrolyte volume (V_a_ = V_c_)	L	1/2
Superficial flow velocity (v_sup_)	cm/s	1
FeSO_4_ concentration in catholyte	g Fe/L as FeSO_4_	0–80
Na_2_SO_4_ concentration in catholyte and anolyte	g Na_2_SO_4_/L	60
H_3_BO_3_ concentration in catholyte and anolyte	g H_3_BO_3_/L	10
Current density (i)	A/m^2^	300
Current (I)	A	3
Plating rate at 100% current efficiency	g Fe/h	3.13
Run time	h	5

**Table 2:** The different polymer mixtures used in the synthesis of the novel anion exchange membranes (AEMs). PVBCl = poly vinylbenzyl chloride; TMIm = 1,2,4,5-tetramethyl-imidazole; F_6_PB = fluorinated polybenzimidazole; PBIOO = polybenzimidazole; SFS = partially fluorinated sulfonated arylene main-chain polymer; SAC = sulfonated poly(phenylethersulfone).

No.	Name	Halogenated Polymer (g)	Tertiary Amine (g)	Polybenzimidazole (PBI) (g)	Sulfonated Polymer (g)	PEG-Diepoxide (g)
1	2243A	PVBCl (0.75)	TMIm (1.22)	F_6_PBI (0.70)	SFS001 (0.18)	PEG500 * (0.10)
2	MHC4-C	PPOBr (1.20)	TMIm (**)	PBIOO (0.80)	SAC098 (0.30)	-
3	2258	PVBCl (2.00)	TMIm (1.30)	F_6_PBI (1.30)	SFS001 (0.35)	PEG500 * (0.30)
4	2281A	PVBCl (2.25)	TMIm (3.66)	PBIOO (2.40)	SFS001 (0.55)	-
5	2285A	PVBCl (2.25)	TMIm (3.66)	PBIOO (2.40)	sPEEKNa (0.62)	-
6	2244B	PVBCl (2.25)	TMIm (3.66)	F_6_PBI (2.40)	SFS001 (0.53)	-
7	2259	PVBCl (2.75)	TMIm (4.50)	F_6_PBI (1.30)	SFS001 (0.35)	PEG500 * (0.30)
8	2244C	PVBCl (1.50)	TMIm (2.44)	F_6_PBI (1.60)	SFS001 (0.35)	-
9	2252A	PVBCl (1.50)	TMIm (2.44)	PBIOO (1.60)	SAC098 (0.43)	-
10	BM-5	PPOBr (0.80)	TMIm (0.65)	PBIOO (1.2)	SAC096 (0.2)	-
11	2408-2	PVBCl (1.50)	TMIm (1.3)	F6PBI (2.35)	-	-

* Sigma-Aldrich product no. 475696, average M_n_ 500 Da. ** membrane was post-treated in TMIm solution (50 wt. % in ethanol) at 60 °C for 2 days.

**Table 3:** Polymer components of the PBI-blended membranes.

Halogenated Polymers		Tertiary Amine
PVBCl	PPOBr	TMIm
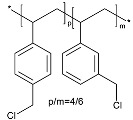	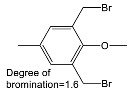	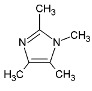
PBI polymers		
F_6_PBI		PBIOO
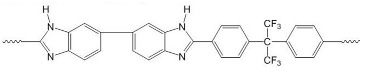		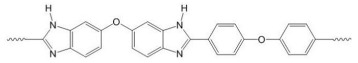
Sulfonated polymers		
SFS		SAC
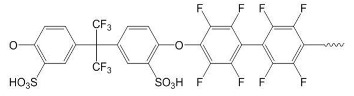		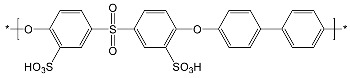
sPEEKNa		
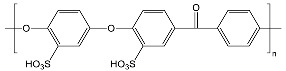		
Poly(ethylene glycol diepoxide)		
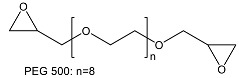		

PVBCl = poly vinylbenzyl chloride; PPOBr = poly(p-phenylene oxide) (PPO) brominated; TMIm = 1,2,4,5-tetramethyl-imidazole; F_6_PB = fluorinated polybenzimidazole; PBIOO = polybenzimidazole; SFS = partially fluorinated sulfonated arylene main-chain polymer; SAC = sulfonated poly(phenylethersulfone); sPEEKNa = sulfonated poly(ether ether ketone).

**Table 4:** Comparison of data obtained after 5 h with the three EW processes.

Variable	Undivided	Terylene Membrane	FAB-PK-130
pH catholyte	2.05	2.90	2.51
pH anolyte	2.05	1.56	0.84
Fe plated (g)	3.30	12.1	15.5
Current efficiency (%)	21	77	99
Specific energy consumption (SEC) (kWh/kg Fe)	17.7	9.50	3.75
[H_2_SO_4_] (g/L)	0.45	1.4	7.2

**Table 5:** IEC_total_, chloride conductivity, and water uptake of the synthesised AEMs.

Membrane	IEC_total_ (meq/g)	$\sigma_{Cl^{-}}$ (mS/cm)	Water Uptake 25 °C/90 °C (%)
(1) 2243A	2.45	10.5	40/50
(2) MHC4-C	-	-	-
(3) 2258	2.42	26.5	77/91
(4) 2281A	2.91	45.5	34/39
(5) 2285A	2.89	4.30	34/39
(6) 2244B	2.50	5.20	37/41
(7) 2259	2.41	22.0	157/258
(8) 2244C	2.50	1.60	27/27
(9) 2252A	2.90	4.60	37/44
(10) BM-5	0.28	0.09	10/4
(11) 2408-2	0.45	0.31	7/5

**Table 6:** Summary of data obtained for novel and commercial membranes during EW.

Name (No.)	FAB-PK-130	2244B (6)	2259 (7)	2244C (8) *	2252A (9) **	BM-5 (10)	2408-2 (11)
pH catholyte	2.51	2.43	2.03	1.89	1.82	2.39	1.9
pH anolyte	0.84	0.98	1.09	0.57	0.99	0.95	0.96
Fe plated (g)	15.5	11.0	11.1	7.8	4.20	14.8	13.8
Efficiency %	99	70	71	83	99	95	88
SEC (kWh/kg Fe)	3.75	5.16	5.13	4.61	3.55	3.53	4.18
Lifetime (h)	>5	>5	>5	3	2	>5	>5

* Values obtained after 3 h. ** Values obtained after 2 h.

## Supplementary Materials

The following are available online at https://www.mdpi.com/2077-0375/9/11/137/s1, Figure S1: XRD analysis of brown precipitate formed during EW in an undivided and porous system. With the analysis showing that the precipitate is goethite/ferric oxyhydroxide; Figure S2: Image of membrane 2243A (1) after use in the EW cell. Which shows that the membrane ruptured during use; Figure S3: Image of membrane MHC4-C (2) after use in the EW cell. Which shows that the membrane ruptured during use; Figure S4: TGA graph of membranes 2258, 2259, 2244B, 2244C, 2252A, 2281A, 2285A. Showing the water loss of the membranes up to a temperature of 200 °C after which degradation started occurring at 250 °C.

## References

[1] Kefeni K.K., Msagati T.A.M., Mamba B.B. (2017). Acid mine drainage: Prevention, treatment options, and resource recovery: A review. J. Clean. Prod..

[2] Johnson D.B., Hallberg K.B. (2005). Acid mine drainage remediation options: A review. Sci. Total Environ..

[3] Crowe C.W., Maddin C.M. (1986). Method for Preventing the Precipitation of Ferric Compounds during the Acid Treatment of Wells. U.S. Patent.

[4] Harris H.J. (1964). Method of Preventing Precipitation of Iron Compounds from an Aqueous Solution. U.S. Patent.

[5] Sharma I.G., Alex P., Bidaye A.C., Suri A.K. (2005). Electrowinning of cobalt from sulphate solutions. Hydrometallurgy.

[6] Jeffrey M.I., Choo W.L., Breuer P.L. (2000). The effect of additives and impurities on the cobalt electrowinning process. Miner. Eng..

[7] Lupi C., Pasquali M. (2003). Electrolytic nickel recovery from lithium-ion batteries. Miner. Eng..

[8] Mostad E., Rolseth S., Thonstad J. (2008). Electrowinning of iron from sulphate solutions. Hydrometallurgy.

[9] Varcoe J.R., Slade R.C. (2005). Prospects for alkaline anion-exchange membranes in low temperature fuel cells. Fuel Cells.

[10] Varcoe J.R., Atanassov P., Dekel D.R., Herring A.M., Hickner M.A., Kohl P.A., Kucernak A.R., Mustain W.E., Nijmeijer K., Scott K. (2014). Anion-exchange membranes in electrochemical energy systems. Energy Environ. Sci..

[11] Arges C.G., Ramani V. (2013). Investigation of Cation Degradation in Anion Exchange Membranes Using Multi-Dimensional NMR Spectroscopy. J. Electrochem. Soc..

[12] Luo J.Y., Wu C.M., Xu T.W., Wu Y.H. (2011). Diffusion dialysis-concept, principle and applications. J. Membr. Sci..

[13] Regel-Rosocka M. (2010). A review on methods of regeneration of spent pickling solutions from steel processing. J. Hazard. Mater..

[14] Agrawal A., Sahu K.K. (2009). An overview of the recovery of acid from spent acidic solutions from steel and electroplating industries. J. Hazard. Mater..

[15] Parasuraman A., Lim T.M., Menictas C., Skyllas-Kazacos M. (2013). Review of material research and development for vanadium redox flow battery applications. Electrochim. Acta.

[16] Sukkar T., Skyllas-Kazacos M. (2004). Membrane stability studies for vanadium redox cell applications. J. Appl. Electrochem..

[17] Chen D., Hickner M.A., Agar E., Kumbur E.C. (2013). Optimized anion exchange membranes for vanadium redox flow batteries. ACS Appl. Mater. Interfaces.

[18] Chen D., Hickner M.A. (2013). V 5+ degradation of sulfonated Radel membranes for vanadium redox flow batteries. Phys. Chem. Chem. Phys..

[19] Tanaka Y., Moon S.-H., Nikonenko V.V., Xu T. (2012). Ion-exchange membranes. Int. J. Chem. Eng..

[20] Carrillo-Abad J., Garcia-Gabaldon M., Ortiz-Gandara I., Bringas E., Urtiaga A.M., Ortiz I., Perez-Herranz V. (2015). Selective recovery of zinc from spent pickling, baths by the combination of membrane-based solvent extraction and electrowinning technologies. Sep. Purif. Technol..

[21] Carrillo-Abad J., Garcia-Gabaldon M., Perez-Herranz V. (2014). Study of the zinc recovery from spent pickling baths by means of an electrochemical membrane reactor using a cation-exchange membrane under galvanostatic control. Sep. Purif. Technol..

[22] Morandi C.G., Peach R., Krieg H.M., Kerres J. (2015). Novel morpholinium-functionalized anion-exchange PBI–polymer blends. J. Mater. Chem. A.

[23] Morandi C.G., Peach R., Krieg H.M., Kerres J. (2015). Novel imidazolium-functionalized anion-exchange polymer PBI blend membranes. J. Membr. Sci..

[24] Kerres J.A., Krieg H.M. (2017). Poly(vinylbenzylchloride) Based Anion-Exchange Blend Membranes (AEBMs): Influence of PEG Additive on Conductivity and Stability. Membranes.

[25] Cho H., Krieg H.M., Kerres J.A. (2018). Application of Novel Anion-Exchange Blend Membranes (AEBMs) to Vanadium Redox Flow Batteries. Membranes.

[26] Mirza A., Burr M., Ellis T., Evans D., Kakengela D., Webb L., Gagnon J., Leclercq F., Johnston A. (2016). Corrosion of lead anodes in base metals electrowinning. J. South. Afr. Inst. Min. Metall..

[27] Tripathy B.C., Singh P., Muir D.M. (2001). Effect of manganese(II) and boric acid on the electrowinning of cobalt from acidic sulfate solutions. Met. Mater. Trans. B.

[28] Jing L., Yang Q.-H., Zhang Z. (2010). Effects of additives on nickel electrowinning from sulfate system. Trans. Nonferrous Met. Soc. China.

[29] Atkins P., de Paula J., Keeler J. (2018). Atkins’ Physical Chemistry.

