# Supplementary material for: Electrowinning of Iron from Spent Leaching Solutions Using Novel Anion Exchange Membranes
Source: Membranes (Basel). 2019 Oct 24;9(11):137. doi: 10.3390/membranes9110137 (PMC6918249; doi:10.3390/membranes9110137)
Supplement: Supplementary file 1 [file membranes-09-00137-s001.pdf]

Article

Supplementary Materials: Electrowinning of Iron from Spent Leaching Solutions Using Novel Anion Exchange Membranes

Wouter Dirk Badenhorst [1], Cloete Rossouw [1], Hyeongrae Cho [2], Jochen Kerres [1,2], Dolf Bruinsma [3] and Henning Krieg [1,*]

[1] Focus Area: Chemical Resource Beneficiation, Faculty of Natural Sciences, North-West University, Potchefstroom 2520, South Africa; 24250643@nwu.ac.za (W.D.B.); 22114688@nwu.ac.za (C.R.); Henning.Krieg@nwu.ac.za (H.K.)

[2] Institute of Chemical Process Engineering, University of Stuttgart, Stuttgart D-70199, Germany; J.Kerres@gmx.de (J.K); hyeongrae.cho@icvt.uni-stuttgart.de (H.C)

[3] Bruinsma Solutions, 10 Mclagen Str., Potchefstroom 2531, South Africa; dolf.bruinsma@gmail.com

* Correspondence: Henning.Krieg@nwu.ac.za

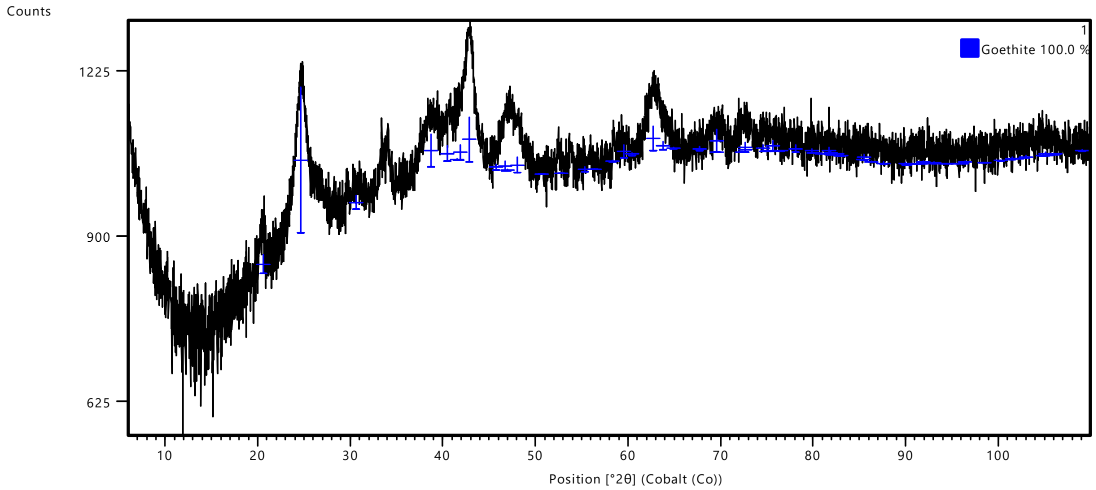

Figure S1. XRD analysis of brown precipitate formed during EW in an undivided and porous system. With the analysis showing that the precipitate is goethite/ferric oxyhydroxide.

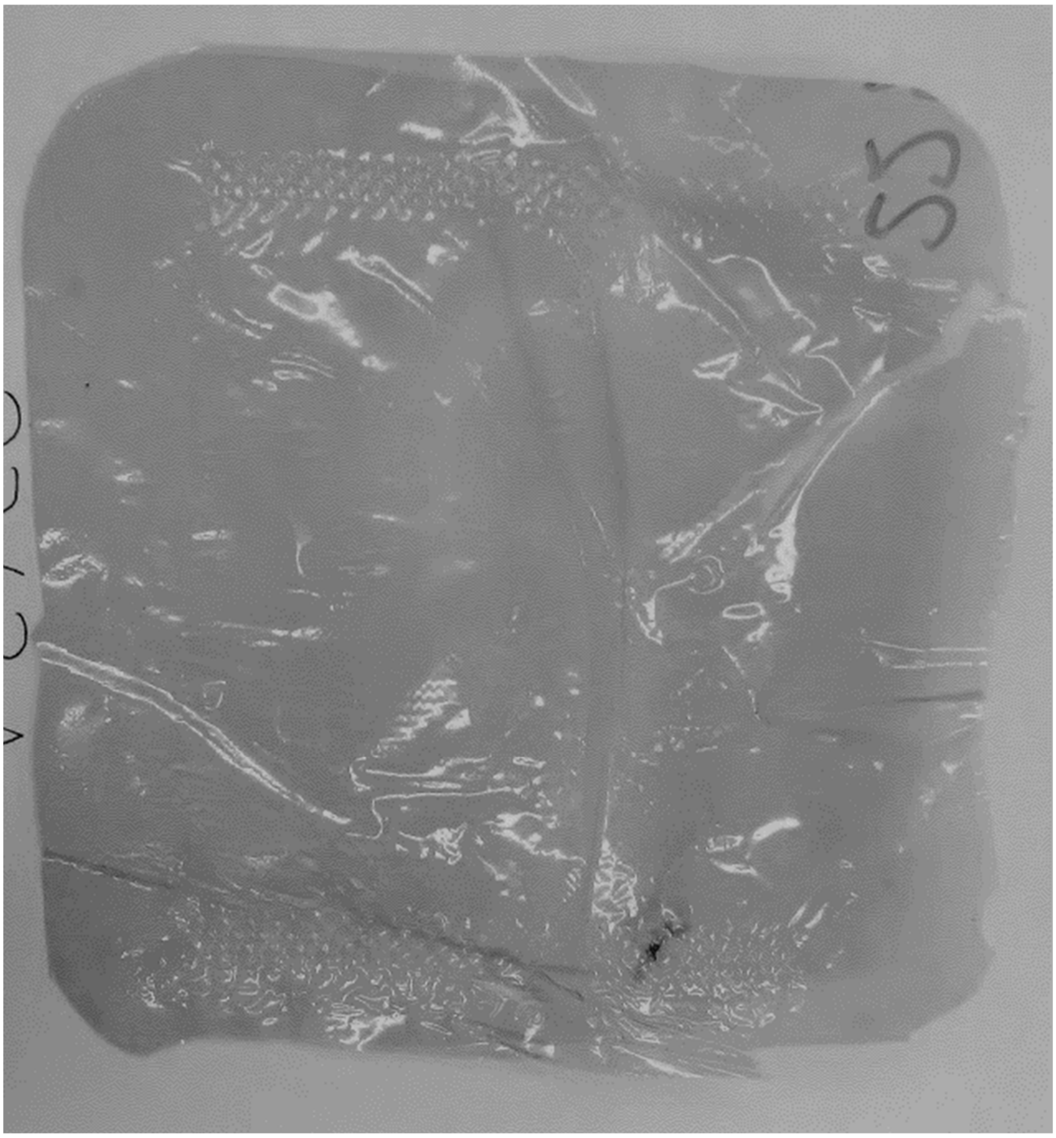

Figure S2. Image of membrane 2243A (1) after use in the EW cell. Which shows that the membrane ruptured during use.

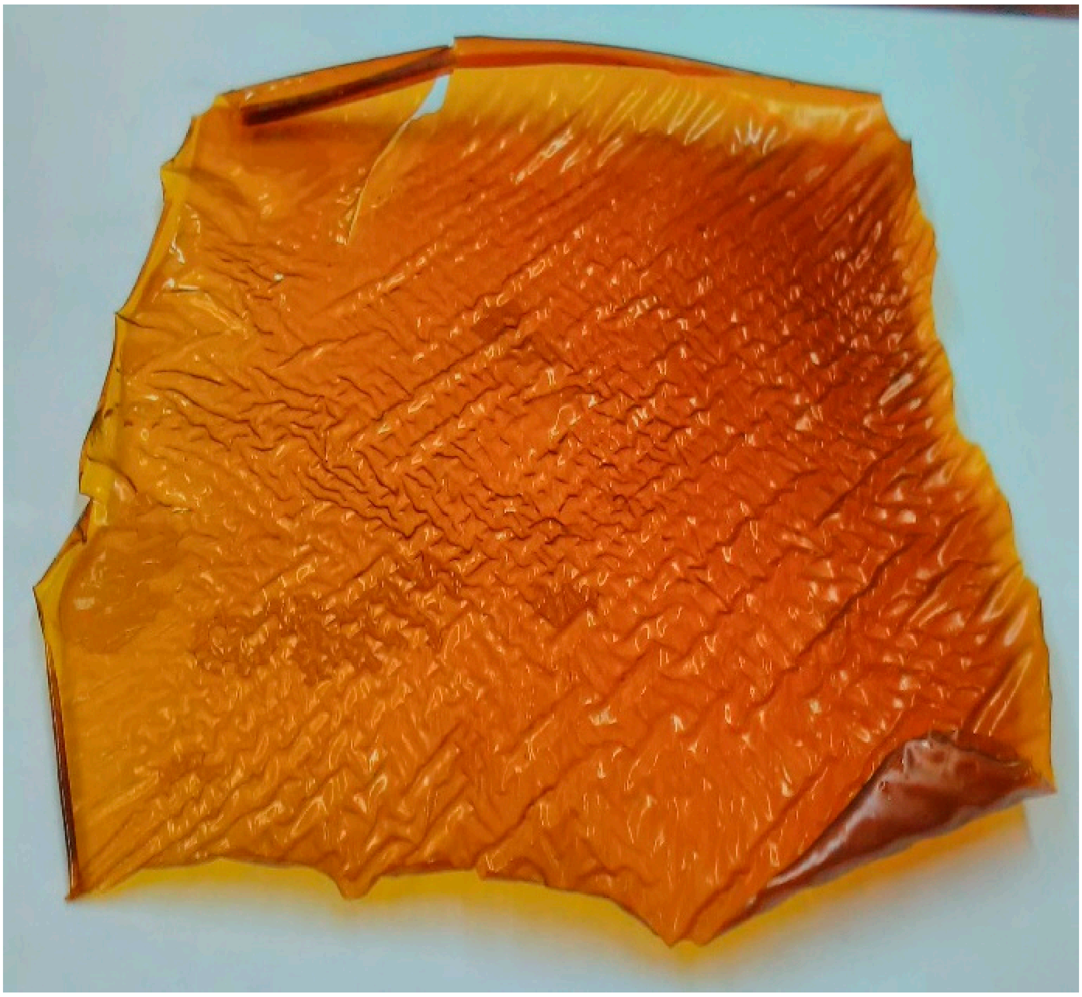

Figure S3. Image of membrane MHC4-C (2) after use in the EW cell. Which shows that the membrane ruptured during use.

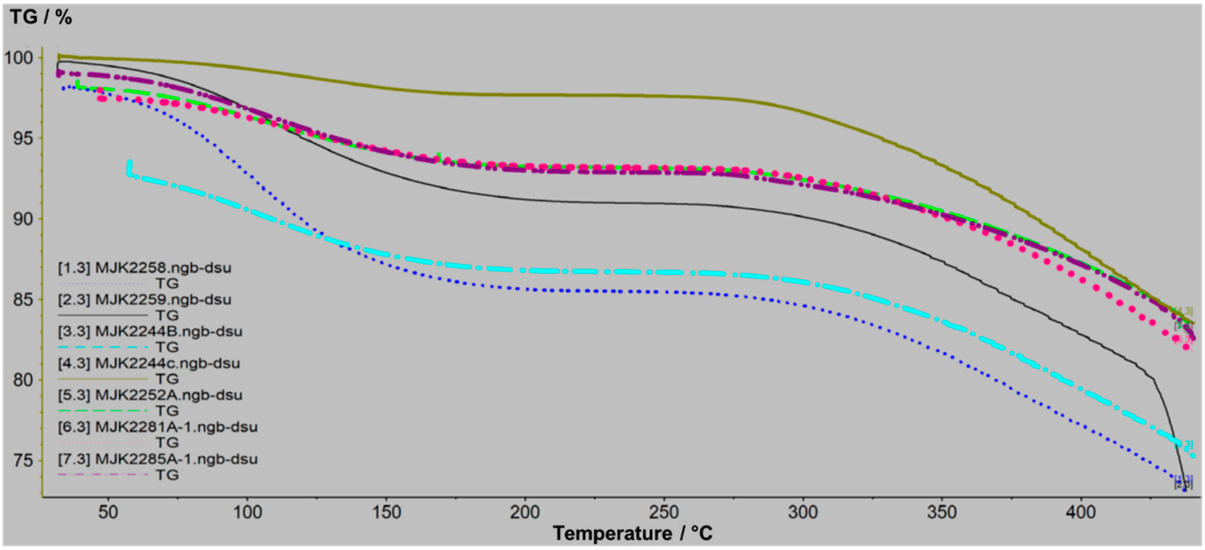

Figure S4. TGA graph of membranes 2258, 2259, 2244B, 2244C, 2252A, 2281A, 2285A. Showing the water loss of the membranes up to a temperature of 200 °C after which degradation started occurring at 250 °C.
